# Ecological effect of the riparian ecosystem in the lower reaches of the Tarim River in northwest China

**DOI:** 10.1371/journal.pone.0208462

**Published:** 2019-01-10

**Authors:** Zulpiya Mamat, Umut Halik, Tayierjiang Aishan, Ayinuer Aini

**Affiliations:** 1 Institute of Arid Ecology and Environment, Xinjiang University, Urumqi, China; 2 Key Laboratory of Oasis Ecology of Ministry of Education, Xinjiang University, Urumqi, China; 3 College of Resource and Environment Sciences, Xinjiang University, Urumqi, China; Universidade Regional Integrada do Alto Uruguai e das Missoes, BRAZIL

## Abstract

The riparian vegetation in the lower reaches of the Tarim River is an irreplaceable natural resource for its ecosystem, and also a guarantee for the transportation safety in this area. Here, we analyzed different plant influences on soil erosion and evaluate the main ecosystem service functions served by the riparian vegetation to study area. Results showed that the total amount of sand-fixation in the study area was 4.14×10^13^ t and that *Tamarix chinensis* had a greater influence on wind speed and sediment transport than *Populus euphratica*, and the *Tamarix chinensis* can be used as suitable vegetation for wind erosion measures and provide scientific basis for the optimization of vegetation matching and reasonable allocation scheme for ecological construction in arid areas. The total ecosystem service value was calculated to be $11.03×10^11^. Of the main ecosystem service functions, riparian vegetation primarily served as sand fixation. Results show that, this research was identical, and the construction of shelterbelt plays an important role in the promotion of wind and sand control measures. Finally, our findings highlights the need for further research on how vegetation function as windbreak and sand fixation.

## Introduction

Ecosystem service valuation is an area of intense focus in ecology as it plays an important role in boosting sustainable ecosystem management and in efficient resource allocation [1, 2]. The capacity of an ecosystem to satisfy human well-being is dependent on its ecosystem functions [3], which include the ecological processes that control the flow of materials, energy, and nutrients in a given ecosystem [4, 5].

Riparian ecosystems are likely to play an essential role in determining the vulnerability of natural and human systems to climate change; moreover, they also influence the capacity of these systems to adapt [6]. The function of a riparian ecosystem depends primarily on the maintenance of a natural hydrologic regime along with adequate biodiversity [7]. Both characteristics are vital for the delivery of provisioning, regulating, and cultural services [5].

While changes to land use/land cover are important in every ecosystem around the world, they are particularly critical in China. This is because land resources per capita are well below the global average [8]. Since China initiated its economic reforms, rapid land-use changes have occurred simultaneously in most of its territories [9]. For example, in the middle reaches of the Tarim River, cropland has been extended apace with increasing population and grain demand. As a result, increasing amounts of runoff from its upper reaches has been intercepted for use in crop irrigation. This has resulted in less runoff entering the lower reaches of the river [10, 11]. The result has been a variety of eco-environmental problems in the lower reaches of the Tarim River, such as river-flow interruptions, drying of terminal lakes, shrinkage of natural vegetation areas, land desertification, intensified sandstorms, soil salinization, and declining water quality [9–13].

The natural riparian vegetation along the Tarim River not only provides ecosystem services for the health and well-being of local people, but must also guarantee the transportation safety of National Highway 218 as well as the current construction of the Golmud–Korla Railway running from Xinjiang to inner China. With the establishment of the “One Belt and One Road” development strategy, the riparian ecosystem of the lower reaches of the Tarim River has now become the primary transportation route for the new Silk Road Economic Belt initiative. Achieving sound ecological status would mean ensuring the ecological integrity of riparian ecosystems like that of the Tarim River, thereby preserving their capacity to provide ecosystem services to humans [7].

Unfortunately, the lower reaches of the Tarim River have been severely degraded due to water shortage caused by agricultural overexploitation of water resources. Since 2000, there has been a sustained effort to recover its riparian vegetation [14–16]. One frequent argument for these restorative measures is the protective function against the soil degradation served by this natural "Green Corridor". Given this, it is logical that the ability to control aeolian sediment is a powerful indicator for the effectiveness of a given restoration project [15]. Therefore, prevention of desertification, and wind-breaking and sand-fixation have become urgent problems faced by the local governments along the lower reaches of the Tarim River.

Here, we assessed the main ecosystem service values of the riparian vegetation of the lower Tarim River using ecosystem service value theory. This quantitative analyses is beneficial to land managers and the general public to gain a better understanding on riparian ecosystem service values. The results obtained from this study will provide a sound scientific basis for policy makers, consumers, and agricultural producers. These results will also reduce and helpful to eliminated the type of short-term economic behavior that has damaging to riparian ecosystem service values and will play an important role in boosting sustainable ecosystem management.

## Materials and methods

### Study area

**Fig 1:**
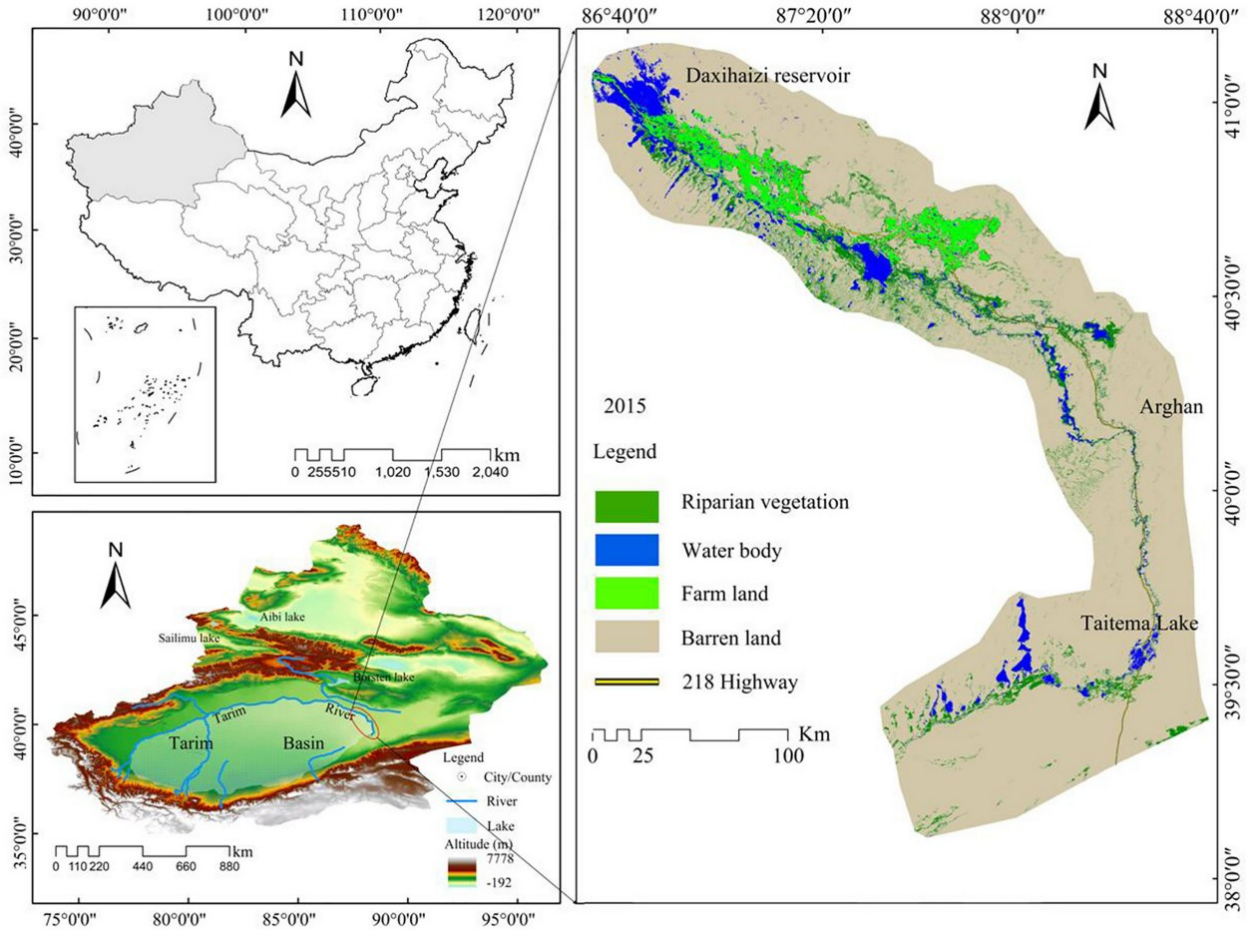
Study region of the lower reaches of the Tarim River.

This study focused on the lower reaches of the Tarim River. The overall length of the lower reaches of the Tarim River from the Chara Reservoir to Taitema Lake is 428 km. The channel bed stretches from north to south on alluvial fans and is located between Taklimakan Desert and Kuruk Desert (39°38′- 41°45′ N, 85°42′- 89° 17′ E) (Fig 1). The lower reaches of the Tarim River are classified as an arid, warm temperate zone. Its annual precipitation varies from 17.4–42.0 mm, and the total annual potential evaporation is approximately 2500–3000 mm. Although this is an arid climate with frequent strong winds, the lower reaches of the Tarim River have been known as a “Green Corridor” due to the wealth of vegetation near the river, and the vegetation types mainly include *Euphrat poplars* (*Populus euphratica Oliv*.), *Tamarix chinensis*, *Phragmites australis* (*Cav*.)*Trin*, *Halimodendron halodendron* (*Pall*.) *Vass*, *glycyrrhiza*. Riverside vegetation provides a natural defense against the wind by obstructing sand movement, and this famous "Green Corridor" has played an important role in keeping National Highway 218 free of obstructions. However, the past 50 years have seen excessive exploitation and unreasonable utilization of water resources in the upper reaches of Tarim River basin. As a result, the channel flow in a 321 km section of the lower reaches stopped in the 1970’s. This has resulted in the drying-up of streams and lakes at the tail of the river. Furthermore, a warming climate has simultaneously led to higher evapotranspiration. Consequently, the natural river course was severely interrupted and the groundwater levels dropped precipitously. This led to the death of a large forested area of Euphrat poplars (*Populus euphratica Oliv*.) that had relied on this groundwater for their survival. Other vegetation in this area has also suffered, resulting in the decline of most shrubs and herbs. The end result of this process has been land desertification and serious damage to the local ecosystem. In order to undue the current desertification and prevent further damage, the ecosystem in the lower reaches of the Tarim River will need to be rehabilitated and protected.

In the research area, the most common soils are: marshy soil, saline soil, poplar soil, residual saline soil, oasis soil and wind-sand soil; the vegetation community in the study area consists of trees, shrubs and herbaceous plants, forming the riverside forest vegetation in arid area, which mainly depends on the influence of hydrology, topography and geological and geomorphological conditions

To this end, the Chinese central government invested $15.56×10^8^ in May 2000 to artificially harnessed the Tarim River through the Ecological Water Conveyance Project (EWCP) to transport water along the river channel from the upper to lower reaches. This process has been continuous, and from 2000 to 2018 there have been 19 times water deliveries from the Daxihaizi Reservoir to the lower reaches of the Tarim River. From the data of monitoring site of Arghan, it showed that the groundwater depth changes from 12.65–9.65 m, and indicating that the response of EWCP is very significant. Under the influence of gradually increasing EWCP, the riparian vegetation in the lower reaches of the Tarim river has been restored to varying degrees, and shows the effect of "replenishing water and returning green".

### Data sources

The data for this study originated from (1) the results of our research team’s 2015 field investigation of the lower reaches of the Tarim River and the research results published and shared database on network [17–20], (2) public figures for the Forest Ecosystem Service Function Evaluation, provided by the Chinese government [17], and (3) land-use/land cover data obtained using ArcView GIS software (v. 10.0). We confirm that this research do not referred to any ethical questions, all necessary permits were obtained for the described field studies in China. The authors have no conflict of the research.

### Calculation methods

#### 1. Sediment fixation

The amount of sediment fixation (G) were obtained using the difference between a model scenario with vegetation (G_veg_) to a reference scenario without vegetation (G_ref_) [21]:

$$G=G_{veg}-G_{ref}$$ (Eq 1)

$$G_{total}=\sum_{i=1}^{n}G(u_{i})\times P_{e}(u_{i})$$ (Eq 2)

Where G(*u*_*i*_) is the sediment fixation for windspeed *u*, *P*_*e*_ is the probability above the threshold.

#### 2. Calculation of the ecosystem service values

(1) The value of economic loss due to transportation were obtained using the shadow engineering method [19], as following equation:

$$U_{3}=G÷\rho \times C$$ (Eq 3)

Where *U*_*3*_ refers to the value of reduced economic losses during transportation ($/year), *p* is the sand bulk density (g/cm^3^), and C is the unit cost for sand cleaning ($/m^3^).

(2) The value of reduced farm land economic loss were obtained using the markt value method [19], as following equation:

$$U_{5}=B\times G÷\left(H\times \rho \times 10000\right)$$ (Eq 4)

Where *U*_*5*_ is the value of economic loss due to reduced farm land ($/year), B is an average farm land return ($/(hm^2^·a)), H is soil layer thickness (m), and *p* is the sand bulk density (g/cm^3^).

(3) The soil fertility value

The value of lost soil nutrients were obtained using the markt value method [22–24], as following equation:

$$U_{1}=G\left(NC_{1}/R_{1}+PC_{1}/R_{2}+KC_{2}/R_{3}+MC_{3}\right)$$ (Eq 5)

Where *U*_*1*_ refers to the fertility maintenance of the forest ($/year). *M* is the soil organic matter (%) and *R*_*1*_, *R*_*2*,_ and *R*_*3*_ are the N, P, K contents (%) of the phosphate amine fertilizer, respectively. C_1,_ C_2,_ C_3_ are the prices ($/t) of the phosphate amine fertilizer, potassium chloride fertilizer, and organic matter, respectively.

(4) Value of CO_2_ fixation and O_2_ release

A forest ecosystem is a more complicated ecological system, as its vegetation engages in carbon dioxide fixation through photosynthesis and the subsequent release of oxygen. In contrast to plant respiration, both litter and soil respiration release CO_2_. Therefore, the forest CO_2_ fixation and O_2_ release value were obtained using the markt value method [25], as following equation:

$$Q\left(t\right)=\left[C\left(t\right)-R_{d}\left(t\right)-R_{s}\left(t\right)\right]\times 12/24\times \mu$$ (Eq 6)

$$Q_{2}=A\times F\times 1.26\times 1.2$$ (Eq 7)

Where *Q* is the value of fixed CO_2_ ($), *C* is the net amount of carbon fixed by plants through photosynthesis in one year (t/(hm^2^·a)), *R*_*d*_ is the amount of carbon from litter respiration (t/(hm^2^·a)), *R*_*s*_ is the amount of carbon from soil respiration (t/(hm^2^·a)), and *u* is the average plantation cost ($/m^3^), *Q*_*2*_ is the value of O_2_ release ($), *A* is the riparian vegetation area (hm^2^), F is the average afforestation cost ($/m^3^).

(5)Value of increasing precipitation

The value of increasing precipitation (P) by using the markt value method [25], as following equation:

P=A×E×10%×0.67 (Eq 8)

Where *A* is the riparian vegetation area (hm^2^) and *E* is the mean annual evapotranspiration (mm/year).

(6) Dust retention value

Natural vegetation in the desert, the value of purifying the atmosphere is primarily a function of dust detaining. Using the product cost method [25], we can estimate the dust retention value as follows:

$$V_{d}=Q_{d}AC_{d}$$ (Eq 9)

Where *V*_*d*_ is the dust-retention value ($), *Q*_*d*_ is the dust retention ability (t/hm^2^), *C*_*d*_ is reducing dust costs ($/t) [26], and *A* is the total area of riparian vegetation (hm^2^).

(7) Absorbing sulfur value

The absorbing SO_2_ value were obtained using the product cost method [27], as following equation:

$$V=\sum_{i=1}^{2}S_{i}\times \left(F_{1}+F_{2}\right)$$ (Eq 10)

Where *V* is the absorbing sulfur value ($), ∑*S*_i_ is the total amount of SO_2_ is absorbed by needle and broad-leaf forest every year (t/year), *F*_*1*_ is the cut the cost of SO_2_ ($/t), *F*_*2*_ is the operating cost ($/t).

(8) Animal habitat value

The value of this animal habitat were obtained using the shadow engineering method [25], as follows:

V=μ×A×5% (Eq 11)

Where *V* is value of the animal habitat assuming ($), *μ* is a cost to build a 1 hm^2^ equivalent zoo.

(9) Value of increasing biodiversity

V=(β+μ)×A (Eq 12)

Where *V* is the value of increasing biodiversity($), *β* is the forest recreation and biodiversity value loss caused by deforestation ($/hm^2^); *μ* is the global willingness to pay for forest products ($/hm^2^) [19].

(10) Water conservation value

The water conservation value was evaluated using the shadow engineering method [28], as following equation:

V=(R−E)×A×P (Eq 13)

Where *V* is the water conservation value, *R* is the average precipitation (m/year), *E* is average evapotranspiration (m/year), *A* is the total area of riparian vegetation (hm^2^), *P* is reservoir construction cost ($/m^2^).

#### 3. Inflation adjusted prices

All commodities prices were adjusted for inflation using 2015 as the reference year [29].

$$V_{2015}=V_{t}\frac{CPI_{2015}}{CPI_{t}}$$ (Eq 14)

Where *V*_*t*_ is the original value in the year t, CPI_2015_ is the 2015 consumer price index (CPI), and CPI_t_ is the CPI of year t.

## Results

### Estimation of sand fixation

Using plants as a method for sand fixation is an effective way to control a desert ecosystem. Sand fixation is also known as soil erosion load and it is dependent on both wind speed and the threshold-controlled character of aeolian sediment transport [15, 16]. Here, the Arghan that located in the lower reaches of Tarim river was selected as our experimental area. The Arghan is located in the lower reaches of the Tarim River and its sand fixation was estimated using our previously described experimental method [21]. Since our selected research area had a relatively flat terrain with an extremely arid climate, the topography and soil moisture had no significant influence on the redistribution of sand grains. Given this, the influence of vegetation on sand transport is extremely significant.

The main conditions that allow for severe wind erosion are extremely dry soil and a relatively stable critical wind speed over the surface [30]. Critical wind speed refers to the minimum speed required for a granular bed to change from a static to a fluid state [30]. According to previous studies, wind speed (4.8 m/s) at a height of 2 m at an observation point was determined to be the critical starting wind speed for soil erosion. Moreover, winds greater than 4.8 m/s were found to be erodible winds [15].

**Fig 2:**
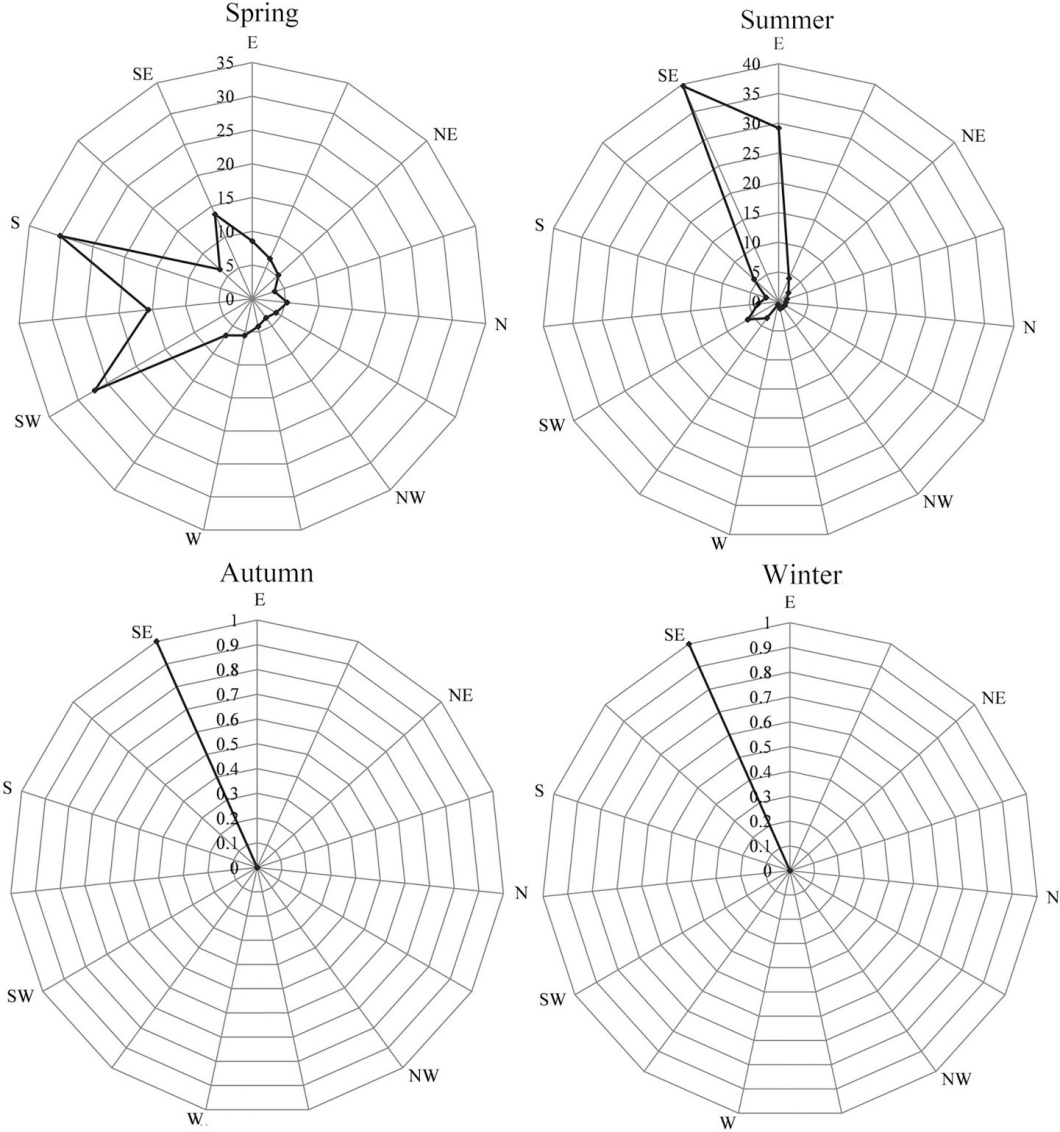
Wind direction across different seasons in the study area.

In our field observation, we found that the main wind direction in the study area in spring was S-SW, where S accounted for 30.14% of all wind direction, SW 27.15%, and SE 13.71%. The main wind direction in summer was SE-E, where SE accounted for 39.67% of all wind direction, E is 29.17%, and SW is 6.07%. The main wind direction in both autumn and winter was SE, with a frequency of 100% (Fig 2). The months with the highest average wind speed were April, May, and June, with average wind speeds of 6.28 m/s, 6.68 m/s, and 5.75 m/s, respectively. August and September had the lowest wind speeds of 0.92 m/s and 0.33 m/s, respectively.

**Fig 3:**
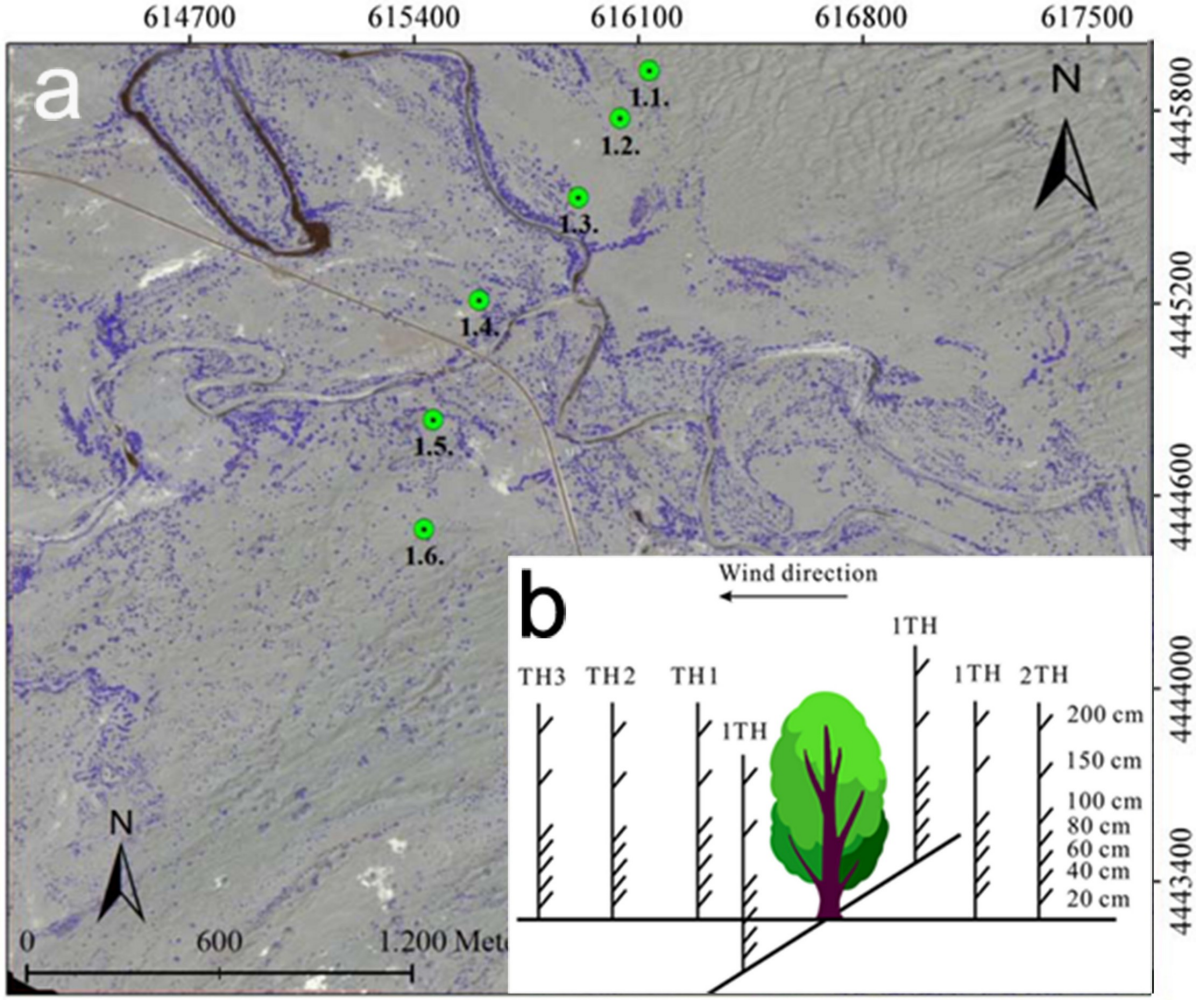
(a) Sediment trap installing points at two transect in the experimental area (Arghan) and (b) Apparatus to observe wind velocity around a single plant.

Given the changes in wind speed and direction in the study area, we selected section to monitor sediment transport that was perpendicular to the direction of the prevailing wind. We started from the Kuruk desert and extended from north to south to the Taklamakan; at each point, our sand collecting instrumentation was arranged at a typical sample point (Fig 3A). Our results revealed that near the river, there was a large shelter effect with almost no exposure of the soil to the wind. As a result, there was no sediment transport—even on bare soil with lower wind speeds (<0.69 m/s). Consequently, there was no sediment fixation afforded by the local vegetation.

Under more adverse condition when wind speed was higher (14.6 m/s), the sediment transport volume for the whole investigation area was equal to 36,672 kg/m∙h. Based on the different sediment retention values, soil erosion amount of the riparian vegetation for the whole experimental area was calculated to be 11,656 kg/m∙h. The total sand fixation for the lower reaches of the Tarim River was also assessed on the basis of previous experimental results from 2015 and the total amount of was calculated to be 4.14×10^13^ t [15].

### Wind-preventing and sand-controlling effects of individual plants

This study used the common tree *Euphrates poplar* and common shrub *Tamarix chinensis* as research objects to assess the wind-preventing and sand-controlling effect of individual plants from two species. Within the study area, we selected a region with a relatively flat terrain that also had severe wind erosion. We then selected three individual *Euphrates poplar* trees (A1, A2, A3) and three individual *Tamarix chinensis* shrubs (B1, B2, B3) (Table 1) and assessed both wind speed and sediment concentration.

**Table 1:** Individual characteristics of a single vegetation on the surrounding landform.

Tree species	Tree height (m)	Crown size (cm)	Crown loss (%)	Comment
**A1**	8.00	650×600	30	East empty land, north, south, west 10–15 m places have *Populus euphratica*
**A2**	7.00	600×400	60	East empty ground, north, south, west 10–15 m places have *Populus euphratica*
**A3**	6.50	400×450	80	There are dead shrubs at 10 m in the east and empty land in the north and south
**B1**	4.5	700×600	20	The topography of the east and northeast is high and empty, and the *tamarix* shrubs are located 50 m west
**B2**	4.00	600×500	50	In the east 30 m, there is dead *tamaricx*, and in the north 20 m, there is *tamaricx*
**B3**	3.50	400×350	80	There are tamarix willow and dead *Halimodendron halodendron* wood 100 m to the east, and the north and south are empty

The main parameters for our wind speed observations were as follows: The anemometer was stabilized at the position of 2TH and 1TH on the windward side, 1TH, 2TH, 3TH, and 4TH on the leeward side, and 1TH and 2TH on the profile of a single plant. On both sides of the selected plants, spots that were 20 cm, 40 cm, 60 cm, 80 cm, 100 cm, 150 cm, 200 cm, 250 cm, and 300 cm perpendicular to the wind direction were chosen. The recording time was set to 1 min for each side (Fig 3B). Installing the sand collecting instrumentation occurred synchronously with the wind speed observation. Instrumentation was installed at 2TH and 1TH on the windward side of a single plant, 1TH and 2TH on its leeward side, and 1TH on its profile(Table 1). Simultaneous measurements were obtained from the bare ground.

**Fig 4:**
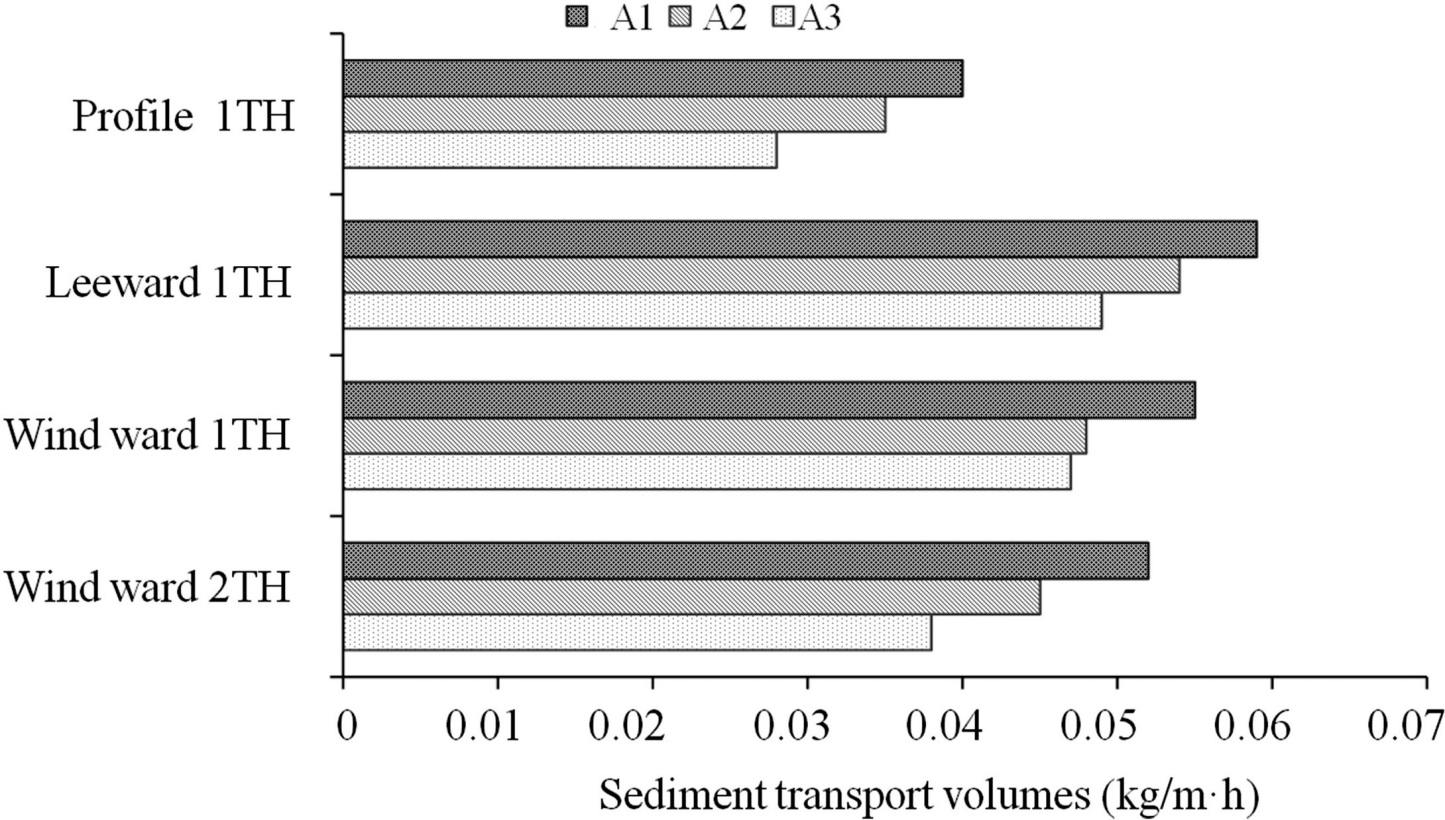
The total sediment discharge of around the three types of *p*. *euphratica*.

A1, A2, and A3 were ranked according to changes of wind speed from large to small at different altitudes (Fig 4). Rankings were as follows: 300 cm > 250 cm > 200 cm > 150 cm > 100 cm > 80 cm > 60 cm > 40 cm > 20 cm. Since wind speed increase with height, the difference in wind speed was greater. Comparative measurements on the bare ground indicated that wind speed on bare ground was higher than that around a single plant.

The wind speed variation at different horizontal distances occurred in the following order: bare ground > profile 1TH > windward 2TH> leeward 2TH> windward 1TH> leeward 1TH > leeward 0.5TH. Of these, the wind speed of 1TH on the profile of A1 was the highest at different heights, while the wind speed at 0.5TH on the leeward surface was the lowest. A2 had the largest change in wind speed at a height of 300 cm; the closer to the ground surface, the smaller the change. A3 had little change in wind speed at 300 cm and 250 cm, but significant difference in wind speeds below 200 cm.

Wind speed measurements at different azimuths for *Populus euphratica* indicated that there were no significant differences between the surface wind speed on its windward and leeward sides; the surface wind speed on either profile of the selected *Populus euphratica* trees was greater than the windward side and the surface wind speed on the leeward side has a was attenuated to a certain degree. However, the degree of attenuation varied with the type of plant and a given plant’s crown loss. Under the same conditions in the study area, the wind speed of A1 at the 20 cm height of the leeward side at 0.5TH as decreased by 58%, and the wind speed at 100 cm height as decreased by 49%. The wind speed of A2 at the 20 cm height of the leeward side at 0.5TH as reduced by 50% and the wind speed at 100 cm height as reduced by 42%. A3 had a reduced wind speed 46% at the 20 cm height of the leeward side at 0.5TH and 37% at 100 cm. The difference in wind resistance between the three poplars was mainly due to the growth of a given poplar. The better an individual’s growth, the more luxuriant the canopy structure. This resulted in smaller the canopy loss and greater wind protection. The worse the growth, the looser the canopy structure, the greater the crown loss, and the smaller the wind-prevention effect. A1 had a small degree of crown loss with vigorous crown growth. A2 had normal crown loss and growth. When compared with A1 and A2, A3 had a large degree of crown loss and poor growth. Given these findings, the wind-prevention effect of A1 was markedly better than for either A2 or A3.

The total sediment transport volume of 1TH on the profile of the three *Populus euphratica* trees was the largest, with sediment transport volumes of 0.049 kg/m∙h, 0.054 kg/m∙h, and 0.059 kg/m.h, respectively. The total sediment transport at 1TH on the leeward side was the smallest, at 0.028 kg/m∙h, 0.035 kg/m∙h, and 0.04 kg/m∙h, respectively. The sediment transport volume at 2TH on the windward side was less than 1TH on the windward side. Given this, the sand-controlling capacity of the three *Populus euphratica* trees were in the following order: A1 > A2 > A3. Notably, this is the same order as the change of wind speed at each observation point for each tree.

**Fig 5:**
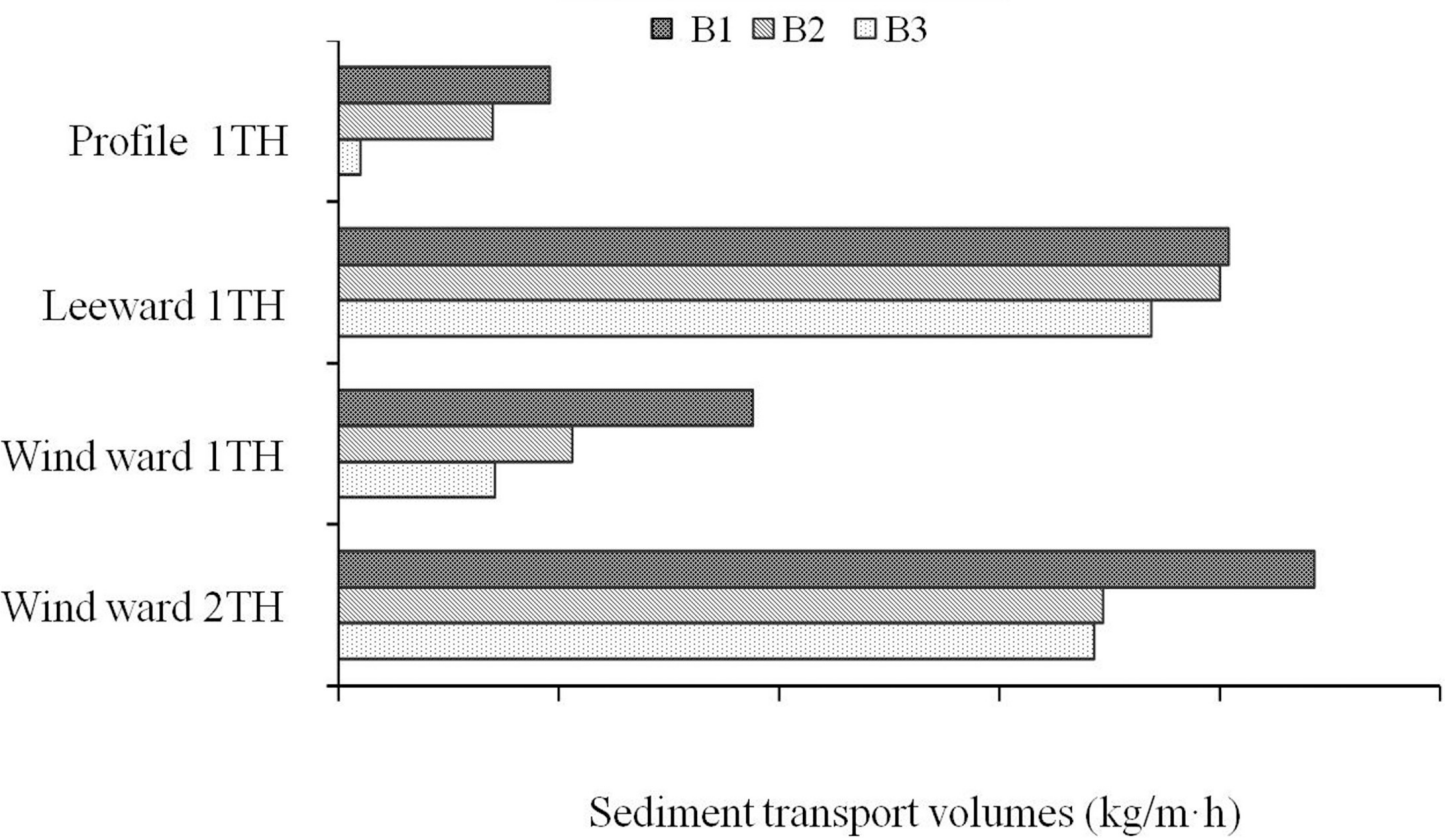
The total sediment discharge of around the three types of *Tamarix spp*.

B1, B2, and B3 were also measured at different heights (Fig 5), with wind speed changes occurring from largest to smallest as follows: 300 cm> 250 cm> 200 cm> 150 cm> 100 cm> 80 cm > 60 cm > 40 cm > 20 cm. As the wind speed increased with increasing height, the difference in wind speed was greater. This resulted in wind speed differences of between 6 and 6.8 times. Bare ground measurements indicated a higher wind speed than when compared with that around a single plant.

The change in wind speed for B1 and B2 at different horizontal distances occurred in the following order: Bare ground > profile 1TH > windward 2TH > leeward 3TH > windward 1TH > leeward 2TH > leeward 1TH. Of these, the wind speed variation of B2 at a height of 150–200 cm was the smallest, while a height of 100–200 cm was the largest. Wind speed change for B3 at different horizontal distances was as follows: Bare ground > windward 2TH > leeward 3TH > profile 1TH > windward 1TH > leeward 2TH> leeward 1TH.

The wind speed changes across different directions for *Tamarix chinensis* indicated that the surface wind speeds on either profile of a *Tamarix chinensis* shrub was greater than that on the windward side. Moreover, the wind speed on the leeward side has a degree of attenuation. Due to the large crown of B1, canopy loss was 20% and the crown size was 700×600 cm. The wind reached the windward side of B1 and the wind speed on windward 1TH at 300 cm was measured at 7.34 m/s. When the wind reached the *Tamarix chinensis* shrub, most of the airflow was forced to rise to form a high speed zone. When the airflow reached the leeward 2TH, the wind speed formed a vortex and decreased rapidly to 2.11 m/s; this resulted in a static area. When it reached the leeward 3TH, the wind speed rose to 6.98 m/s and recovered most of its original windward wind speed. The canopy loss of B2 was 45% and the crown size was 600×500 cm. When airflow went to leeward 1TH, wind speed decreased from 6.99 m/s to 4.01 m/s; however, this decrease was less than that seen in B1. The reason for this is the greater canopy loss, the less energy the airflow expends as it passes through the shrub. As a result, there is less velocity decay. In addition, the change in the wind speed range on the profile of the shrub was not as notable as B1. This indicated that the greater the canopy size, the more significant the wind speed attenuation on the profile of the plant. B3 had the largest degree of canopy loss, and its growth was not as good as either B1 or B2. Given these findings, the overall wind-prevention order was as follows: B1 > B2 > B3.

The total sediment transport volume of the three *Tamarix chinensis* shrubs (B1-B3) at profile 1TH was the largest, with sediment transport volumes of 0.049 kg/m∙h, 0.054 kg/m∙h, and 0.059 kg/m∙h, respectively. The total sediment transport volume at the leeward 1TH was the smallest, with values of 0.01 kg/m∙h, 0.07 kg/m∙h, and 0.096 kg/m∙h, respectively. Given this, the corresponding sand-controlling effects of each shrub were: B1 > B2 > B3.

Given the results of both studies, it is clear that the *Populus euphratica* trees had no obvious effect on either the surface wind speed or sediment transport. In contrast, the influence of *Tamarix chinensis* shrubs on both wind speed and sediment transport was much more significant, this research was consistent with the results from lower reaches of Heihe river by Han et al., in 2011[31].

### Assessment of riparian ecosystem service values

By combining the Physical Assessment Method (PAM) with the Value Assessment Method (VAM) [32], the contributions of each ecosystem function to the overall ecosystem service values provided by the riparian vegetation in the lower reaches of the Tarim River were assessed (Table 2). The total ecosystem service value of the riparian vegetation in the lower reaches of the Tarim River was calculated to be $11.03×10^11^. The individual contributions of each ecosystem function to the total service value were quite different. Of the ecosystem functions values, the contribution of reducing economical losses in transportation value was the highest (>99%), reducing farm land economy loss value was less than 0.14%, and then increasing precipitation value was the least, at 0.0002%.

**Table 2:** 2015 ecosystem service values of the lower reaches of the Tarim River.

Ecosystem service function	Total ($)
**Soil fertility**	6.58×10^7^
**Reducing economical losses in transportation**	1.10×10^12^
**Reducing farm land economy loss**	15.28×10^8^
**Value of CO**_**2**_ **fixation and O**_**2**_ **release**	26.5×10^6^
**Increasing precipitation**	38.31×10^5^
**Dust retention value**	1.43×10^8^
**Absorbing sulfur value**	7.26×10^6^
**Water** **conservation**	1.53×10^7^
**Dust detaining**	2.48×10^8^
**Animal habitat**	2.75×10^8^
**Increasing biodiversity**	5.13×10^8^

Given these findings, it is clear that the reducing economical losses in transportation value and reducing farm land economy loss value were remained highly significant contributors to the whole riparian ecosystem services value. This is because the riparian ecosystem—along with the Tarim River and National Highway 218—is a large corridor that crosses the middle of the desert. This region in northwest China is a region of highly frequent dust storms [14]. However, riparian vegetation can absorb enormous amounts of dust in the air above by producing mucilage secretion and oils [30]. This natural function will be beneficial to improving China's highway management system as well as promoting the transportation industry between Xinjiang Uygur Autonomous Region and the Chinese Mainland.

The ecosystem of the lower reaches of the Tarim River is a simple riparian ecosystem and is made up of only a few species, including *Populus euphrates* and *Tamarix chinensis*. According to some research, the flora in this region consists of 14 families, 24 genera, and about 40 species of vascular plants [33]. The Tarim River cuts through a large desert, with infrequent rainfall and a severe environment. As a result, the plant species found here form a natural community that needs to endure extreme droughts, and play a crucial role in withstanding the destructive natural forces like sand storms. The Tarim River is also home to more than 15 species of mammals, 51 kinds of birds, 16 species of amphibians, and several kinds of fish. Since 1999, China introduced a widespread logging ban in the Nature Reserve, which is found in the natural forests in the lower reaches of Tarim River. As a result of this effective protection, this area has been allowed to begin to restore itself and has seen the reemergence of several species of wild animals that had not been seen for years.

## Discussion

Since ecosystem services are often difficult to evaluate directly, indirect valuation techniques are generally required to obtain location specific values. The “Green Corridor” of the lower reaches of the Tarim River along with National Highway 218 often parallel to the desert ecosystem and plays a vital function in preventing the closure of the Taklamakan and Kuruk deserts and connection of desert oases.

Here, we combined the sand fixation model [21] with field survey results to estimate the soil wind erosion in a study area of the lower reaches of the Tarim River. In the survey, we found that the effect of vegetation in different distribution patterns on the redistribution of sediment was different. The vegetation in the study area was mainly distributed in two patterns. One pattern was a sparse, random distribution in the desert that often formed a braid after high winds. Given the distance between these sparse distributions, they are not connected to the surrounding sand braid. As a result, the wind direction of the study area is not fixed, so the azimuths of the braid will also changes with a change in wind direction.

The results of the wind prevention and sand-controlling effect of vegetation with different canopy loss indicated the following order: B1 > A1 > B2 > B3 > A2 > A3. These results are closely related to the canopy loss, plant height, and canopy width. In addition, shrubs closer to the ground had denser branches and leaves, resulting in better coverage. As *Populus euphratica* is a tree, it is higher off the ground and has smaller ground coverage. This results in an overall poorer protective effect.

The riparian vegetation ecosystem service functions values in the study area were monetized using ecological economic methods; these were made on the basis of interviews in 2015 with local experts, farmers, and staff in Ruoqiang and Yuli counties along with “LY/T 172–2008 Evaluation Standard” [17]. Of the main ecosystem functions, sand fixation value was the highest, followed by reducing farm land economy loss value. These results corroborated actual regional situations, demonstrating the validity of this approach and its applicability in this field. It also highlights the need for further research on windbreak and sand fixation effect. We also found a trend of decreasing sediment fixation with increasing distance to the river. This was due to the lower vegetation coverage in these areas. This result is also in good agreement with findings from other studies that showed the duration and volume of water delivery is closely related to the restoration of riparian vegetation in the lower reaches of the Tarim River [16, 30, 32].

Over the past five decades, population density and agricultural intensification have increased, leading to rapid land use and land cover changes in the upper and middle reaches of the Tarim River. These changes have been the main causes of subsequent ecosystem changes including water shortage, vegetation retrogression, sandstorms, and desertification [8, 10, 33, 34]. Further contributing to these negative effects was the construction of the Daxihaizi Reservoir in 1972, which dried up a 321 km river way in the lower reaches of the river. This lack of surface water replenishment led to a precipitous drop in the groundwater table to 8–12 m for nearly 30 years. This led to significant ecosystem degeneration and the large-scale death of natural vegetation. Large areas of farmland were overwhelmed by sand and native animal populations were forced to relocate.

As another significant part of the Silk Road, the Korla (the Korla-Ruoqiang section in Xinjiang Uygur Autonomous Region, approximately 708 km) and Golmud (Ruoqiang- Golmud section in Qinghai Province, approximately 504 km) railroad construction followed along the green corridor in the lower reaches of the Tarim River. This project was started in 2014 and it is a transit corridor as well as energy pathway between China, west Asia, the Mediterranean, and the Black Sea regions. The Korla-Ruoqiang section is one of the districts facing the most serious desertification and the sand fixation function of its riparian vegetation is an ecological barrier to sandstorm disasters. The continued presence of this vegetation will play an important role in the future development of this area’s regional economy.

The presence and length of high sand dunes stretching to the transit corridor along with the region’s strong wind activity will seriously affect the safe operation of current and future highway and railway lines. If not properly accounted for, there is even the potential for serious accidents. Therefore, there is a great need for additional research on windbreak and sand fixation functions. Moreover, there is a need for basic national policies in China regarding railway construction that will allow for both environmental protection and the implementation of a sustainable development strategy.

## Conclusion

Here, we used a 2015 field survey date and sand fixation model to calculate the amount of sand fixation in the study area selected on the Tarim River. Results showed that:

(1) The total amount of sand-fixation in the lower reaches of the Tarim River was 4.14×10^13^ t. The influence of *Tamarix chinensis* on wind speed and sand fixation was significantly more important than that of *Populus euphratica*.

(2) Indirect economic valuation methods were used to quantify the ecosystem service function values of riparian vegetation. The total ecosystem service value of the riparian vegetation in the study area was $11.03×10^11^. Of the main ecosystem functions, the contribution of sand fixation value was highest, followed by reducing farm land economy loss value.

(3) Collectively, this research showed that the riparian vegetation in this area served as an ecological barrier to the impact of sandstorms. Given this, it is critically important to better understand the desert ecosystem function value for their future protection, management, and rational utilization.
