# Correction: Ecological effect of the riparian ecosystem in the lower reaches of the Tarim River in northwest China

**DOI:** 10.1371/journal.pone.0214007

**Published:** 2019-03-14

**Authors:** Zulpiya Mamat, Umut Halik, Tayierjiang Aishan, Ayinuer Aini

The first author, Zulpiya Mamat, is incorrectly noted as the corresponding author. The correct corresponding author is Umut Halik. Dr. Halik’s email address is: halik@xju.edu.cn.

There are errors in the Author Contributions. The correct contributions are: Data curation: UH, TA, AA. Funding acquisition: ZM, UH, TA. Investigation: UH, AA. Project administration: UH, ZM. Resources: UH.

There are errors in the Funding statement. The correct Funding statement is as follows: This study was supported by the National Natural Science Foundation of China (Nos. U1703102, 31860134, 31700386), the Natural Science Foundation of Xinjiang (No. 2018D01C065) and the Thousand Youth Talents Plan of China: Xinjiang Projects. The funders had no role in study design, data collection and analysis, decision to publish, or preparation of the manuscript.
